# Killing Mechanisms of Chimeric Antigen Receptor (CAR) T Cells

**DOI:** 10.3390/ijms20061283

**Published:** 2019-03-14

**Authors:** Mohamed-Reda Benmebarek, Clara Helke Karches, Bruno Loureiro Cadilha, Stefanie Lesch, Stefan Endres, Sebastian Kobold

**Affiliations:** Center of Integrated Protein Science Munich (CIPS-M) and Division of Clinical Pharmacology, Department of Medicine IV, University Hospital, Ludwig-Maximilians-Universität München, Member of the German Center for Lung Research (DZL), 80337 Munich, Germany; MohamedReda.Benmebarek@med.uni-muenchen.de (M.-R.B.); clara.karches@web.de (C.H.K.); bruno.cadilha@med.uni-muenchen.de (B.L.C.); steffi-lesch@t-online.de (S.L.); endres@lmu.de (S.E.)

**Keywords:** chimeric antigen receptor, adoptive T cell therapy, cancer immunotherapy

## Abstract

Effective adoptive T cell therapy (ACT) comprises the killing of cancer cells through the therapeutic use of transferred T cells. One of the main ACT approaches is chimeric antigen receptor (CAR) T cell therapy. CAR T cells mediate MHC-unrestricted tumor cell killing by enabling T cells to bind target cell surface antigens through a single-chain variable fragment (scFv) recognition domain. Upon engagement, CAR T cells form a non-classical immune synapse (IS), required for their effector function. These cells then mediate their anti-tumoral effects through the perforin and granzyme axis, the Fas and Fas ligand axis, as well as the release of cytokines to sensitize the tumor stroma. Their persistence in the host and functional outputs are tightly dependent on the receptor’s individual components—scFv, spacer domain, and costimulatory domains—and how said component functions converge to augment CAR T cell performance. In this review, we bring forth the successes and limitations of CAR T cell therapy. We delve further into the current understanding of how CAR T cells are designed to function, survive, and ultimately mediate their anti-tumoral effects.

## 1. Introduction

Adoptive T cell therapy (ACT) describes the therapeutic use of T cells [1,2]. 

Stemming from the idea that tumor-specific T cells could eradicate cancer, three independent ACT approaches were developed. Chronologically, tumor infiltrating lymphocytes (TIL) were the first approach to be tried, relying on the harvest of T cells directly from a tumor, followed by ex vivo expansion, activation, and finally, patient reinfusion. Limited access to resectable metastases or tumors, time-consuming T cell preparation, and scarce tumor-reactive T cell clones have so far hindered this strategy’s success [3,4]. Current ongoing phase III clinical trials might however shed light on the value of this strategy in melanoma (NCT00200577) [5]. Secondly, T cells genetically engineered with T cell receptor (TCR) started being generated to tackle some major pitfalls of TIL therapy. Through viral transduction, high amounts of peripheral blood mononuclear T cells (PBMC) could be genetically modified to be tumor specific through recognition of major histocompatibility complex (MHC)-restricted peptides. This specificity remains inherently restricted because of its dependence on antigens expressed by tumors via their MHC complexes [6]. The third ACT approach to reach the spotlight consists of the so-called chimeric antigen receptor (CAR) modified T cells, gaining an edge over the previous two with an ingenious series of modifications [7,8].

**Figure 1:**
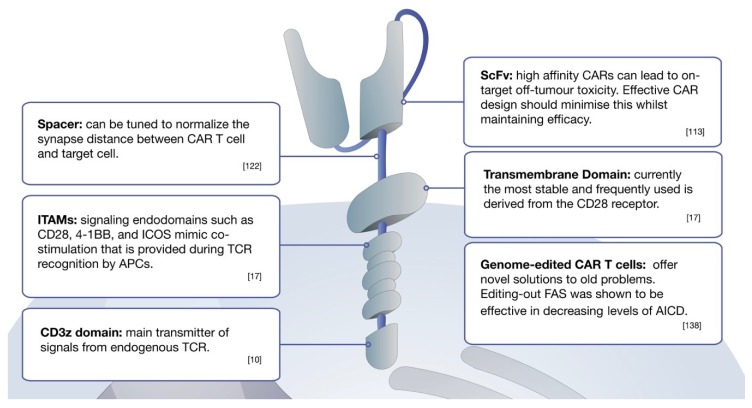
A chimeric antigen receptor (CAR) is composed of several components, each of which contributes towards the proper activation, functionality, and persistence of CAR T cells. In addition to the CAR, T cell gene editing approaches can also augment functional potential.

A CAR is a synthetic construct that can bind to target cell surface antigens through a single-chain variable fragment (scFv) recognition domain, as depicted in Figure 1 [9]. The initial concept linked this ligand recognition domain to an intracellular signaling module composed of a portion of the cluster of differentiation(CD)-3 zeta (3ζ) chain to induce T cell activation upon antigen binding [10,11]. These two modules are connected through an extracellular hinge domain and a transmembrane domain, forming the simplest form of a CAR, currently referred to as a first-generation CAR. The TCR CD3ζ chain contains 3 immuno-tyrosine activation motifs (ITAMs) [12], thus, this chain alone can deliver a potent signal 1 in the absence of other components from the TCR-CD3 complex (the γ, δ and ε chains) [13,14]. Signaling is initiated by lymphocyte-specific protein tyrosine kinase (Lck)-mediated phosphorylation of ITAMs within the cytoplasmic domain of CD3. Soon thereafter, efforts to improve the existing CAR molecule led to the rise of second and third generation CAR architectures that incorporated signaling endodomains, such as CD28, CD137 (also known as 4-1BB), and inducible T cell co-stimulator (ICOS), in an attempt to mimic the co-stimulation that is provided during TCR recognition by antigen presenting cells (APCs) [15,16,17]. This co-stimulatory signal, propagated by phosphoinositide 3-kinase PI3K (in the case of CD28) [18,19,20], is required for full physiological T cell activation [21]. Further developments into fourth or fifth generation CAR T cells included signaling domains from cytokine receptors or inducible expression of inflammatory cytokines, such as interleukin-12 (IL-12) or IL-18 [22,23].

CAR T cells, unlike conventional effector T cells, can recognize antigens irrespective of MHC presentation, nevertheless being limited to the recognition of surface expressed structures. [6,24]. Like TCR engineered T cells, CAR T cells can also be generated upon viral transduction of PBMC and expanded to several orders of magnitude before being administered into a patient. Therefore, this therapy can be generated in an autologous fashion [25].

Unparalleled clinical efficacy has been demonstrated using anti-CD19-CAR T cells to treat refractory CD19^+^ B cell malignancies [15,26,27,28]. A phase I dose escalation study of CD19 CAR therapy showed durable remissions in children. Of the 55 pediatric patients treated, 93% reached complete response (CR) (88% minimal residual disease (MRD) negative) [29,30]. At a median follow up of 1 year, CR was observed in 34 patients. Of those, 20 subsequently relapsed, 13 of them with CD19^−^ disease (antigen-loss-driven disease relapse). Two therapies (Kymriah™ and Yescarta™) were recently approved by the Food and Drug Administration (FDA). Yescarta™ (axicabtagene ciloleucel) is used to treat adults with relapsed or refractory (r/r) large B-cell lymphoma, while Kymriah™ (tisagenlecleucel) is for the treatment of pediatric patients with B-cell precursor acute lymphoblastic leukemia (ALL). 

The clinical efficacy of tisagenlecleucel in r/r B-cell ALL patients was evaluated in an open-label multicenter single-arm trial (88 patients enrolled, 68 treated, 63 available for efficacy). 52 patients had CR and were (MRD) negative within 3 months following infusion. The median duration of the response was not yet estimable [31]. Axicabtagene ciloleucel, when evaluated in an open-label multicenter single-arm trial, also showed significant efficacy. Of the 101 patients treated, 73 had an objective response (52 had CR; 21 had partial remission (PR)). Median time to response was 0.9 months, with median duration of the response lasting 9.2 months [32,33].

In spite of these successes, most of the patients will not benefit in the long run, and current strategies need to address key issues surrounding the loss of therapeutic effectiveness in hematologic cancers, as well as a feeble response in solid tumors and treatment-related toxicities [34].

Clinical studies of anti-CD19 CAR T cells have showcased disease relapse to be most frequently antigen negative [35]. This stresses the concept that antigen loss should be anticipated in the design of future therapies. A phase I study [36] revealed that anti-CD22 CAR T cells could mediate disease remission in B-ALL that is naive or resistant to anti-CD19 CAR T cell immunotherapy, with complete remission observed in 5/5 patients with CD19^dim^/CD19^−^ B-ALL. They have also shown that bispecific CAR T cells targeting both CD19 and CD22 can recognize and kill CD19^+^CD22^+^, CD19^−^CD22^+^, and CD19^+^CD22^−^ B-ALL, pointing towards a strategy able to overcome anti-CD19 CAR T cell limitations [37].

Target selection is critical, beyond therapy effectiveness, for safety purposes. Several reports describe on-target off-tumor activity as a major pitfall during pre-clinical and clinical CAR T cell therapy development [38,39,40,41]. Nevertheless, toxicities associated with CAR T cells are mostly on-target off-tumor, the spectrum of which is dependent upon the affinity and specificity of scFvs, and the activation status of the T cells. Unlike off-target effects of cytotoxic chemotherapy that can result in irreversible genetic modifications [42], CAR T cell toxicities should be reversible upon target cell elimination, or T cell depletion if required [43,44]. 

Excessive T cell activation has been shown to propagate, via monocytes, an IL-1 and IL-6 driven cytokine release syndrome (CRS) [45,46]. Tocilizumab is an anti-interleukin-6-receptor antagonist that has been successful in the management of CAR T cell mediated CRS [27,47]. Despite improvements in the management of associated toxicities (mainly glucocorticoids and IL-6 receptor blockade) [30], a great disparity remains in how patients respond. It is important to stress that, although CAR T cells constitute an approved treatment, we only have a limited understanding of their mode of action regarding both therapy and side effects. It will be important to foster a greater insight into the mechanistic understanding and molecular interplay of a treatment modality that has been fast-tracked to the forefront of cancer therapy [48].

The aim of this review is to convey the current understanding of the mechanisms employed by CAR T cells to mediate their anti-tumoral effects. In line with this, we outline the various aspects that must be considered during effective CAR design that translate into the production of an effective and durable killer T cell that might outperform other ACT modalities.

## 2. Non-Classical Immune Synapse Formation

Conventional cytotoxic T lymphocytes (CTLs) rapidly destroy and eliminate their target cell with remarkable specificity, due to the formation of a distinct immunological synapse (IS) upon engagement of the TCR [49]. This highly organized structure is comprised of a series of concentric rings (supramolecular activating clusters (SMAC)), each of which originates from clustered molecules conferring specific functions. The central SMAC (cSMAC), composed of the TCR and Lck clusters, enhances and amplifies the lethal response through the accumulation of T cell activating signals and the delivery of cytotoxic granules. It is surrounded by the peripheral SMAC (pSMAC), a ring of lymphocyte function associated antigen-1 (LFA-1) adhesion molecules that stabilizes both the IS and target cell binding. The distal SMAC (dSMAC), an aggregation of actin, completes the bull’s-eye structure of the IS [50]. 

**Figure 2:**
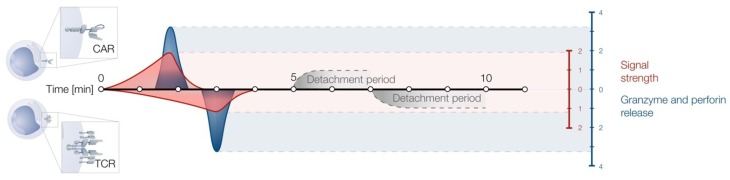
CAR vs T cell receptor (TCR) T cell functionality: Time interval between synapse formation and disengagement following lysis is shorter for CAR T cells compared to TCR T cells. Signal strength during engagement is stronger in CAR T cells compared to TCR T cells. Quantified granzyme and perforin release during engagement was also comparable, despite the difference kinetics. Units are depicted relative to fold change. Granzyme and perforin release depicted in blue. Signal strength depicted in red. (Adapted from [51]).

It is known that CAR T cells utilize, at least in part, the conventional TCR signaling machinery [21]. Thus, one could assume a CAR T cell-target cell IS to be comparable to the classical one. Contrarily, Davenport and colleagues revealed substantial alterations in the IS structure formed by a CAR in comparison to the classical TCR IS. Whilst demonstrating that LFA-1 is dispensable for the IS formation of both receptors, the CAR IS lacks the clustering of Lck within the cSMAC and shows a more disordered pattern of Lck micro-patches. Consequently, the inner diameter of the CAR IS was found to be significantly smaller in size, correlating with faster CAR T cell detachment. Furthermore, a large-scale reverse-phase protein array identified rapid down regulation of the proximal signaling protein protein kinase C-delta (PKCδ) in CAR T cells, suggesting a shorter CAR-initiated signaling duration. These findings were supported by an accelerated delivery of lytic granules to the IS, resulting in faster killing of the target cells [51]. A comparison of CAR and TCR immune synapse activity is depicted in Figure 2. 

In addition, Xiong et al. developed a new strategy to predict the effectiveness of CAR T cells measured by the quality of the CAR-mediated IS. Here, the quantification of F-actin, clustering of tumor antigen, polarization of lytic granules and distribution of essential signaling molecules within the IS comprise the key determinant factors. They could predict superior CAR T cell functionality utilizing a 4-1BB co-stimulatory domain by evaluating the composition of the IS, confirming their findings in vitro and in vivo [52].

The formation of a stable IS is the main prerequisite for the induction of target cell killing by T cells. Once the IS has been formed, tumor cell lysis can be induced by the effector cell utilizing different pathways. 

## 3. Perforin and Granzyme

To mediate cytolytic effector functions, T cells predominantly make use of two major pathways: exocytosis of cytotoxic granules containing perforin and granzymes, and the expression of membrane bound tumor necrosis factor (TNF) family ligands, inducing target cell apoptosis upon engagement with their respective receptor. These two pathways can be subdivided into slow-acting (TNF family ligands) and fast-acting (degranulation) killing mechanisms [53].

To ensure the fast and precise killing of an infected or a malignant target cell, cytotoxic granules are anchored to the microtubules of the effector cell. Upon formation of the immunological synapse, the granules migrate towards the interface and fuse to the plasma membrane within the area of the cSMAC [54]. The vesicles with their cytolytic payload are released into the synaptic cleft, where perforin induces pore formation on the target cell membrane to facilitate the access of pro-apoptotic granzymes. Once in the cytoplasm of the target cell, granzymes can induce caspase dependent and independent apoptotic cell death by cleaving their key substrates [55,56]. 

In the murine system, however, CD8^+^ cytotoxic T lymphocytes (CTLs) rely on granule exocytosis and CD4^+^ CTLs resort to the Fas and Fas ligand (FasL) pathway to mediate cytotoxicity [57,58], whereas in the human system, CTLs (both CD8^+^ and CD4^+^) predominantly utilize the cytolytic perforin and granzyme axis to mediate target cell apoptosis [59]. In addition, when equipped with a CAR, human T cells of both subsets can effectively eradicate tumor cells in a MHC- and Fas-independent manner [60,61]. Therefore, the cytolytic degranulation of perforin and granzymes is assumed to be the main mechanism of redirected target cell killing exerted by CAR T cells [62,63,64]. Blocking perforin release via egtazic acid (EGTA), a calcium ion chelator, was shown to abrogate most CAR T cell-mediated killing [65].

CD4^+^ T cells are reported to express substantially lower amounts of intracellular perforin and granzymes compared to CD8^+^ T cells. Accordingly, effective target cell killing by CD4^+^ CAR T cells is either delayed or requires higher numbers of effector cells to achieve comparable cytolysis to CD8^+^ CAR T cells [61,66]. 

Interestingly, failure of perforin- and granzyme-mediated cytotoxicity by CTLs greatly prolongs the duration of the IS. Blocking caspase processing in the target cell demonstrated that T cell disengagement was specifically dependent on target cell death, which provides a caspase-dependent signal for detachment [67]. Without the timely detachment of the T cell from its target, repetitive calcium signaling, and the augmented hypersecretion of inflammatory cytokines and chemokines that accompanies it, this could result in IL-6 secretion via the activation of naïve macrophages (something that has been shown to be differentially required for CAR T cell related cytokine release syndrome and neurotoxicity) [45]. It remains to be elucidated whether this holds true for CAR T cell mechanisms of action and their related toxicities.

The perforin and granzyme pathways are pivotal for rapid, effective, and specific CAR T cell-induced target cell lysis. This mode of action truly relies on the expression of tumor associated antigens without further need for death receptor molecules presented by the tumor. Concerns have been raised as to whether tumor cells can circumvent elimination by the immune system via the expression of TNF family ligands, thus counterattacking infiltrating lymphocytes [68,69].

It has been previously shown that perforin and FasL CTL killing mechanisms can collaborate. However, FasL-mediated action is typically delayed in this scenario as a result of the early pre-lytic processes induced in the target cell [70]. As will be discussed, synergistic or additive effects between degranulation and ligand-based lytic pathways have been shown to occur in CAR T cells. In particular, FasL can facilitate lytic action even when degranulation is poor or hampered [70,71]. This synergy could be important for the induction of complete and durable tumor control by CAR T cells. 

## 4. Fas and Fas Ligand (FasL) Axis

Classically involved in immune cell homeostasis in non-pathogenic situations, the Fas and FasL pathway has been shown to be multifunctional, both to the benefit and detriment of effector T cells [69,72]. Together with calcium dependent granule exocytosis [51,73], calcium independent Fas and FasL killing is a major axis by which target cells are lysed by T cells [74,75,76]. Recent reports have shown that CAR T cells have the capacity to utilize this pathway to mediate tumor killing.

The Fas and FasL pathway is led by the trimerization of the Fas receptor by Fas ligand [77]. This results in the activation of caspase 8 (mediated by the adapter protein Fas-associated death domain (FADD) and pro-caspase 8, which form the death-inducing signaling complex (DISC)) [72,78]. Active caspase 8 is then responsible for the processing of downstream pro-caspase 3 to form mature caspase 3, which goes on to mediate cell death via the subsequent cleavage of more than 500 cellular substrates, effectively executing the apoptosis program [79,80].

A study by Hong et al. demonstrated that activated CD30 and CD19 targeting CAR T cells (in a tumor milieu containing antigen positive as well as antigen negative tumor cells) were able to mediate tumor lysis against the antigen negative fraction in an antigen independent, cell–cell contact-mediated manner. This lytic mechanism was only observed following CAR T cell activation as a result of their interaction with the antigen positive fraction. Their findings were not observed when CAR T cells were co-cultured solely with the antigen negative fraction. Further, they could demonstrate that FasL was upregulated in CAR T cells following receptor engagement, and that CD30 expression was present on the target cells. In addition, the knockdown of CD95 in the target cells reduced caspase 3 activity compared to wildtype cells following co-culture with antigen-specific CAR T cells. To investigate the broader application of this finding, other tumor cell lines were scrutinized, and it was found that ectopic Fas expression on tumor cells improves CAR T cell activity [81].

Taken together, the Fas and FasL axis constitutes an alternative mechanistic pathway by which CAR T cells can mediate tumor cell lysis within a heterogeneous tumor environment. Antigen-independent bystander killing mechanisms offer a path that could be exploited in the setting of antigen-loss associated disease relapse. In addition, as the challenge of treatment-related toxicity remains unresolved, taking advantage of this pathway could help to overcome complications associated with failed perforin- and granzyme-mediated cytotoxicity.

## 5. Cytokine Production

**Figure 3:**
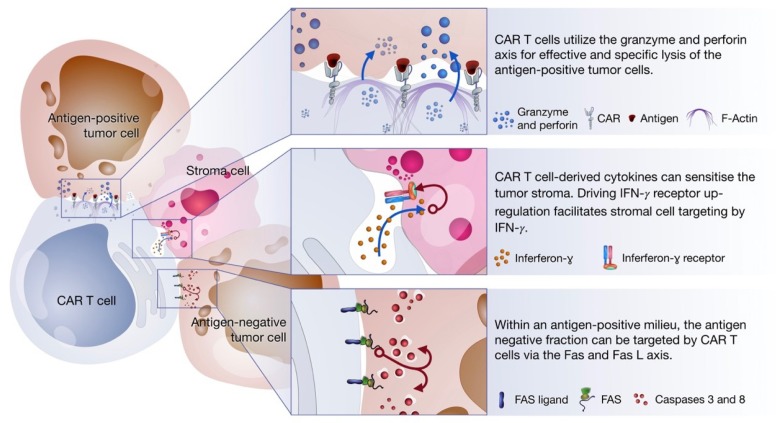
CAR T cells mediate tumor killing via three axes: (1) Perforin and granzyme axis: Targeting antigen positive fraction. (2) Cytokine secretion: Stromal cell sensitization. (3) Fas and FasL axis: Targeting antigen-negative fraction.

While CAR T cell design is based on achieving tumor lysis via direct T cell-tumor cell interactions, cytokine production by activated CAR T cells could further enhance their anti-tumoral capabilities. The fact that solid tumor lesions possess great phenotypic diversity is undoubtedly challenging for a living therapy that relies on a highly specific and targeted approach. Cytokine secretion by CAR T cells plays an important role in mediating tumor lysis via secondary mechanisms [82], as depicted in Figure 3. 

It has been shown that HER-2-specific CAR T cell-derived cytokines could induce interferon gamma (IFN-ɣ) receptor expression by the tumor stroma, whilst driving immune cell re-education (such as the polarization of macrophages towards an anti-tumoral M1 phenotype) [83]. In addition to antigen-specific tumor cell targeting, CAR T cell driven antigen-independent stroma destruction highlights an additional mode of action of CAR T cells. Similarly, natural killer (NK) cell depletion from tumors with induced IFN-ɣ receptor expression did not impede tumor rejection. This further indicates that stromal sensitization and macrophage polarization are key elements supporting CAR T cell mediated tumor cell killing, leading to overall tumor rejection.

When trying to tackle an evolving tumor, one must forecast tumoral evolution as a result of treatment. A multi-functional CAR, whether it is bispecific, switchable, or capable of delivering other therapeutic agents within the tumor, can mediate several effector mechanisms simultaneously, thus offering greater therapeutic potential in solid tumors [36,84,85].

One such multifunctional system is the employment of T cells redirected for universal cytokine killing (TRUCKs). These are CAR T cells used as vehicles to secrete and mediate the accumulation of effector cytokines within the tumor tissue. This approach allows for the controlled and site-directed delivery of effector molecules within the tumor tissue [86], circumventing the problems encountered as a result of their systemic delivery [87]. 

A molecule with pleiotropic functions, IL-12, has been reported to galvanize anti-tumor immune responses [88,89]. Mechanisms involve the amelioration of T cell cytolytic activity [22], the recruitment and activation of innate immune cells [90], and the reprogramming of stroma-associated immune suppressor cells [91]. The TRUCK approach [92,93], which relies on the delivery of IL-12 via CAR-redirected T cells, achieves this whilst simultaneously targeting tumor-associated antigens in an MHC-unrestricted manner [94].

The effectiveness of cytokines in driving anti-tumor immunity is shown not least through the suppression of various cytokine pathways to effectively evade immune surveillance [95]. It is worth noting that dampening IFN-ɣ signaling predominantly results in minimized antigen presentation to CD8^+^ T cells via MHC-I—not reducing the potential cytostatic effects of this cytokine [96]. Supporting this, CAR T cell evasion has been observed to occur primarily through the loss of targeted tumor associated antigens [97,98], rather than any alterations on the IFN-ɣ pathway, for instance [95]. 

Taking this into consideration, one cannot overlook the likely emergence of alternative immune evasion mechanisms. Tumor immune evasion has been shown to occur through the suppression of antigen presentation and cytokine signaling, namely IFN-ɣ and TNF signaling [99,100]. As multifunctional CAR T cell therapies begin to emerge, other immune cell subsets will be brought into play. While this could bring about improved therapeutic benefits, it is likely that immune evasion mechanisms will grow in complexity.

## 6. CAR T Cells as Serial Killers

Ideally, ACT would mediate the rapid destruction of a tumor mass with relatively low numbers of effector cells, ensuring better control of side effects. The ability to sequentially kill multiple target cells has been demonstrated for natural cytotoxic lymphocytes, such as NK and CD8^+^ T cells [101]. Likewise, CAR T cell potential for serial killing has recently been validated [63].

A study by Davenport and colleagues utilized a novel transgenic mouse model to investigate variance in the recognition and killing of target cells through TCR or CAR engagement. CTLs constitutively co-expressing the OT-1 TCR and a CAR specific for HER-2 were generated by cross breeding the respective transgenic mouse strains. TCR-driven T cell stimulation, as well as functional potential (measured in levels of cytotoxic granules), were unaffected by the simultaneous expression of the CAR. Furthermore, equally effective target cell lysis could be observed following CAR or TCR engagement, proving the comparable functionality of both receptors. Live-cell microscopy was employed to compare the kinetics of attachment, recognition, and killing by TCR or CAR T cells. The duration of the time-interval between target recognition and lytic granule delivery was unchanged between CAR and TCR. This functional comparability was present despite the lower surface expression of the CAR [63].

Interestingly, the time interval between T cell-target recognition, and disengagement following lysis (synapse formation) was observed to be shorter lasting in CAR T cells. The signal strength during this period of engagement was observed to be stronger in CAR T cells [51]. However, the frequency of serial killing events was equal for the engagement of both receptors, demonstrating that CAR T cells are in no way inferior to TCR T cells in their sequential killing potential [63].

Another important aspect is the proportion of CD8^+^ and CD4^+^ T cells within the CAR T cell product. The absence of CD4^+^ T helper cells can lead to CD8^+^ T cell dysfunction and apoptosis [102]. Through the incorporation of CD4^+^ T cells into treatment protocols, their importance in the delivery of effective immunotherapies has become apparent [103]. The importance of the interplay between these two cell types, on a kinetic and mechanistic level, was studied by Liadi and colleagues through the observation of longitudinal interactions between anti-CD19 CAR CD8^+^ and CD4^+^ CAR T cells [66]. Single cell analysis revealed equal tumor cell killing by CD4^+^ and CD8^+^ CAR T cells, despite the former doing so following a longer conjunction period and delayed kinetics. 

Furthermore, the authors could subdivide the T cell mode of action into multiplexed killing and serial killing. Multiplexed killing describes the simultaneous engagement with two or more targets. Their results showed both killing mechanisms to be utilized with an equal frequency, although multiplexed killing was seen to be favored at higher tumor densities. Once more, CD4^+^ CAR T cells required longer conjugation for efficient killing of tumor cells regardless of the mechanism employed. These findings could be attributed to the lower intracellular granzyme B content of CD4^+^ T cells and was confirmed by a substantial reduction in tumor cell killing when granule exocytosis was blocked using EGTA [104]. Interestingly, CD4^+^ CAR T cells were less susceptible to activation-induced cell death (AICD) than CD8^+^ CAR T cells [66].

Overall, it is clear that the potential of CD4^+^ CAR T cells to mediate multiple target cell killing could further potentiate the efficacy of CAR T cell therapy. Moving forward, there is a need to identify and deliver optimal CAR T cell subset compositions, an area which is already in focus for current clinical research [105].

Our present understanding of the mechanistic potential of CAR T cells, though limited, has shown them to be adaptable killers capable of targeting tumor cells in various ways. This potential could only be exploited, however, following CAR design that carefully considers its performance, functionality, and persistence within the context of its precise application.

## 7. Affinity Variations of CAR Design Can Maximize Killing Efficiency

scFvs are highly specific, can be readily generated against most antigens of interest, and incorporated with ease into the highly modular CAR design [106]. A drawback of using scFvs is the increased probability for oligomerization, which can lead to tonic signaling [107,108]. 

Many CAR T cell approaches utilize scFvs with high affinities. This rationale was based on previous studies showing CAR T cell activation thresholds to be inversely correlated with scFv affinity. Studies looking at TCR stimulation identified there to be a window of affinity ideal for T cell activation, as further TCR affinity augmentation did not improve treatment efficacy [109,110].

Chmielewski and colleagues have previously shown that high affinity CAR T cells exhibit less discrimination between target cells with high or low target expression levels [111]. Further, anti-Her2 CAR T cells with high affinity led to serious toxicity, due to on-target off-tumor recognition on normal cardiopulmonary tissue [112].

This off-tumor activity can be tuned down via affinity modulation. Liu and colleagues found that decreasing the scFv affinity could significantly increase the therapeutic window of CAR T cells whilst retaining robust anti-tumor efficacy (in vitro and in xenogeneic mouse tumor models). They demonstrated this in anti-HER-2 and anti-epidermal growth factor receptor (EGFR) CAR T cells by reducing the dissociation constant of the scFv domain of CAR T cells by 2 to 3 log. CAR T cells with lower affinity scFvs showed equally durable anti-tumor activity against ErbB2 overexpressing tumors as compared to high affinity CAR T cells, while on-target off-tumor reactivity was significantly reduced [113]. 

CAR T cell approaches have a higher limit (several orders of magnitude) of target sensitivity compared to antibody or antibody-drug-conjugate therapies [114]. With so few tumor targets exclusively expressed at the tumor site [115], affinity modulation will need to be utilized more effectively for safer and more-controlled CAR T cell therapies.

Disparities in reports do exist. Studies showcasing high affinity CAR T cells to be non-reactive to low off-tumor expression have been described [111]. It is important to recognize that, despite their apparent simple design, CAR T cells can have great variability, while targeting the same antigen through different epitopes, for example [116]. Signaling domain configurations, spacer length [117], and gene transfer approaches that could impact CAR expression levels on the T cell surface must be considered [118,119]. A multivariate analysis of both the CAR and its target must be employed when selecting the affinity of a CAR for clinical application.

While scFv affinity is clearly important for both CAR functionality and augmented killing potential, other constituents of CAR design must also be considered in order to optimize functionality. 

## 8. Optimizing CAR T Cell Functionality and Killing Potential

As previously mentioned, CAR T cells do not conform to the classical T cell-target cell plasma membrane permissiveness for assembly of a SMAC [51]. Their inability to conform to this dimensional relationship results from several variables: The target-molecule’s structural dimensions, the epitope location on the target molecule, and the CAR’s spacer length [120]. Despite the target’s molecular rigidity, the spacer length can be tuned to somewhat normalize the synapse distance between CAR T cells and target cells [121]. 

Kunkele and colleagues demonstrated that CAR T cell performance and functionality was highly dependent on both extracellular spacer length and cytoplasmic signaling module selection. In vitro, some of these spacer variations correlated with augmented cytolytic activity and pro-inflammatory cytokine production. Nonetheless, these augmentations led to in vivo hyperactive signaling outputs that correlated strongly with high levels of FasL expression, subsequently resulting in higher levels of AICD [122].

They further investigated whether anti-tumoral activity could be augmented in CAR T cells with an optimized ‘short spacer’ by modulation of endodomains. Second and third generation CAR T cells were directly compared. Findings revealed that, despite improved cytolytic activity and cytokine production, the third generation CAR T cells were more susceptible to AICD as a result of increased FasL surface expression [122,123]. 

CAR T cells can be designed to potentiate their anti-tumoral efficiency [124]. For instance, CAR T cells designed to secrete a checkpoint blocking scFv were shown to protect the CAR T cells from an immunosuppressive microenvironment [125]. Further, CAR T cells have been engineered to co-express immune-regulatory factors. By simultaneously expressing these factors, CAR T cells could not only induce an antigen-specific anti-tumoral response, but were also able to increase tumor infiltration by dendritic cells and other T cells [126]. 

Engaging alternative signaling pathways for CAR T cell mediated killing proved very effective in a pre-clinical study. Kagoya and colleagues [82] demonstrated a CAR construct designed to activate the janus kinase-signal transducer and activator of transcription (JAK-STAT) pathway. They could engage STAT5 by integrating a truncated IL-2 receptor beta (IL2RB) chain, in addition to STAT3 engagement via the addition of a YXXQ motif at the C-terminus of CD3z. Their CAR triggered IL-21 treatment-like gene expression profiles that armed T cells with a unique functionality that included an ameliorated proliferative capacity and superior effector functions, as compared to CD28-CD3z and 4-1BB-CD3z CAR T-cells.

Recently, these differences were further explored, as phosphoproteomic analysis revealed kinetic and quantitative differences resulting in functional divergence [127]. In a disseminated lymphoma xenograft model, a CD28-CD3z CAR showed increased basal phosphorylation of the CAR CD3z chain and CAR associated Lck, leading to greater kinetics and signal strength, which correlated with an effector T cell–like phenotype and function. This signal intensity was partly related to constitutive association of Lck with this domain in CAR complexes. In contrast, 4-1BB-CD3z CAR T cells preferentially expressed T cell memory-associated genes and exhibited sustained anti-tumor activity against established tumors in vivo. 

A preclinical study [16] showcased how ICOS incorporation, as opposed to 4-1BB or CD28, could change T cell fate from a T_H_1 to a follicular T_H_17 cell. Their phenotypically distinct CAR could mediate efficient anti-tumor responses, as well as improved persistence compared to CD28 and 4-1BB-based CAR T cells. A third-generation CAR combining ICOS and 4-1BB was later developed, and showcased superior anti-tumor efficacy against solid tumor models when compared to 4-1BB alone [128]. This and various other approaches employed to regulate and enhance CAR T cell activity are summarized in Table 1 [129,130]. The optimizations that can be made to the CAR molecule itself are an ongoing learning process in the field. As new formats of recognition domains or costimulatory domains come into play, the previous optimal parameters might need to be revised.

## 9. CAR Delivery and Genetic Modifications of CAR T Cells

CAR T cell gene editing processes require the efficient delivery of the coding DNA. Considerations must be made when choosing the optimal carrier, with regards to safety and efficiency. Retroviral or lentiviral infection of lymphocytes are the most commonly used approaches, as they result in T cells with excellent transduction efficiencies [138,139]. However, these approaches entail a tedious manufacturing process and harbor the risk of insertional mutagenesis, which by nature is not predictable and can have negligible, negative, and eventually also beneficial effects [140]. The alternative to viral delivery systems are the non-viral transposon systems PiggyBac and Sleeping Beauty that use the simple “cut and paste” transposase mechanism to integrate the CAR cDNA into the host genome [141]. With less good manufacturing practice (GMP) restrictions, reduced risk for insertional mutagenesis, and lower overall costs, the transposon systems are already superior to viral gene delivery in terms of the procedure of T cell editing, despite their more limited efficiency. The use of minicircle DNA further improved the transposon gene delivery system, with clinical trials using Sleeping Beauty-generated CAR T cells currently ongoing [142,143].

**Figure 4:**
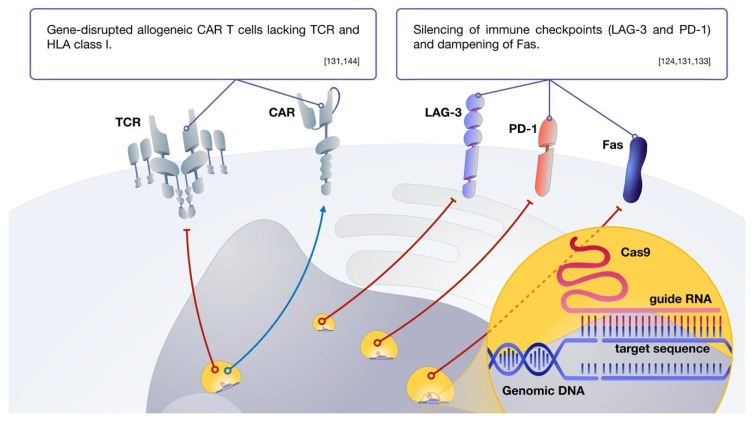
CRISPR-based approaches for the genetic modification of CAR T cells. Gene disruption approaches have been shown to be effective for the silencing of inhibitory axis, and the development of universal CAR T cells. Red solid and dotted lines depict silencing or disruption of genes. Blue line depicts insertion of CAR.

Besides the CAR molecule, a CAR T cell still burdens the whole T cell genomic program and akin signaling cascades. Considering this, genome-editing techniques based on CRISPR technology have started being validated for T cell production. CRISPR, short for clustered regularly interspaced short palindromic repeats, is a genome editing method. Due to its scalability, affordability, and ease of use, it has become the gold-standard method for genome editing [144]. Ren and colleagues have validated a one-shot protocol that generates CAR T cells while concomitantly taking advantage of CRISPR technology to mediate disruption of multiple gene targets. Within this work, they have generated CAR T cells deficient in Fas (to reduce AICD and resist apoptosis), endogenous TCR, and human leukocyte antigen (HLA) (to achieve an allogenic universal cellular product) [131]. The approach of silencing of immune checkpoints via gene editing has proven quite promising. One molecule targeted by CRISPR-Cas9 has been PD-1, leading to enhanced PD-L1^+^ and PD-L2^+^ tumor clearance in vivo [132]. Another negative regulator of T cell activity effectively targeted by CRISPR-Cas9 is lymphocyte activation gene-3 (LAG-3) [133]. The insertion of the CAR cDNA itself can be done under the tight regulation of CRISPR technology to simultaneously disrupt a locus or place the CAR under the control of a specific promoter [120,145]. Multiplex genome editing will thus grow into becoming an even more valuable tool to enhance CAR T cell killing potential as it broadens editing potential beyond the CAR molecule. Some of these approaches are depicted in Figure 4.

Other gene editing approaches, such as transcription activator-like effector nucleases (TALENs) and Zinc-finger nucleases (ZFNs), have seen significant advances and are now being employed for the genetic engineering of T cells [146]. ZFNs are modular repeats fused to bind contiguous DNA sequences. They are made by fusing zinc finger DNA-binding domains to DNA-cleavage domains and can induce double strand breaks to activate DNA damage response pathways, thus allowing specific alterations to be made [147]. TALENs use TALE proteins to function in a similar fashion to ZFNs, whilst also possessing inherent binding specificities, allowing them to be directed towards very specific genomic sites [148,149].

Universal “off-the-shelf” CAR T cells generated from allogeneic donors are currently being developed [85,150]. To the end of allogeneic combination immunotherapy, TALEN was used to disrupt TCRαβ surface expression in CAR T cells to give them multidrug resistance, which is crucial to pre-conditioning lymphodepleting regimens (purine and pyramidine nucleoside analogues such as clofarabine). ZFNs have been used to abrogate the expression of the endogenous TCR and disrupt HLA-A2 in CAR T cells, in further efforts towards the generation of allogenic universal cellular products. [134,150,151].

## 10. Conclusions and Future Perspectives 

Thus far, CAR T cells have been transformative in the treatment of hematological diseases and are rightly regarded as one of the major breakthroughs in cancer immunotherapy. With few exceptions, solid tumors have been barely susceptible to CAR T cell therapy [152]. Mechanistically, this biology remains relatively unexplored, as numerous preclinical and clinical studies seek to elucidate a more refined understanding of CAR T cell functionality.

Powerful techniques, such as live-cell microscopy and single cell analysis, have allowed for the prediction of CAR T cell effectiveness prior to their in vivo characterization. Further, their underlying mode of action, classically believed to be solely dependent on the delivery of lytic granules, has been shown to be more flexible. CAR T cells have also been proven to be more susceptible to apoptosis-inducing surface molecules than has previously been assumed [81,122]. Novel CAR designs offer much in terms of the future potential of the therapy, as efforts are directed towards improving safety and efficiency. The emergence of inducible, switchable, or split CAR T cells, such as the synNotch CAR [125], UniCAR [136], or the split, universal and programmable (SUPRA) CAR [85], have enabled superior flexibility, specificity, and controllability of the therapy [137]. 

Eventually, and despite the continued improvements to the CAR molecule itself, other aspects will need to be considered for the optimization of T cell therapies, such as tweaks to improve migration, infiltration, and to overcome immunosuppression [153,154,155,156]. For instance, the incorporation of antibodies targeting checkpoint inhibition is an approach that is gaining momentum [126,157]. Soon, pre-clinical studies that will rely on approaches to bypass the aforementioned antibodies to achieve the same effect through the genetic modification of T cells might be impactful to the way T cell therapies are developed [158]. Likewise, chemokines have also been deemed as important targets for the improvement of T cell infiltration. Multiple approaches have been developed to target chemokine ligands for neutralization [159], or by modifying T cells to overexpress chemokine receptors whose cognate ligands are expressed in the tumor micro-environment [160,161]. 

The road towards the broader and more successful application of CAR T cell therapy is currently being paved. CAR T cell functionality, specificity, and efficiency are continually improved. In combination with advances in cell engineering and gene editing, CAR T cells have yet unmet potential in cancer treatment. The recent approval of two CAR T cell therapies marks the dawn of a new era in cell therapy, where broad applicability of such approaches will need to be demonstrated.

## Figures and Tables

**Table 1:** Summary of the various approaches employed to regulate CAR T cell activity, and to enhance their selectivity and killing potential.

Aim	Modulation	Approaches
Enhancing selectivity	Simultaneous targeting of multiple antigens	Tandem CAR [130]
		iCAR (inhibitory CAR) [129]
	scFv modulation	Fine-tune scFv affinities [113]
	Inducible CARs	synNotch CAR [124]
Enhancing killing potential	Co-stimulatory domains	JAK-STAT CAR [82]
		3rd generation ICOS CAR [128]
	Cytokine production	TRUCK system [86]
	Checkpoint Blockade	Secretion of PD-1 scFv [125]
	Targeted delivery of CAR cDNA to disrupt a locus	CRIPSR guided to Fas, endogenous TCR, PD-1, and LAG-3 [131,132,133]
		TALEN-mediated multi-drug resistant CARs [134]
	Immune cell recruitment	7 × 19 CAR (co-expressing IL-7 and CCL 19) [126]
Regulating activity	Suicide Gene	Inducible Caspase9 [135]
		Antibody-mediated depletion via marker antigen [43]
	Switchable CAR	Tumor targeting anti- or nanobody (UniCAR, SUPRA CAR) [85,136]
		Dimerization through small molecules [137]
